# Mitochondria and Inflammation: Cell Death Heats Up

**DOI:** 10.3389/fcell.2019.00100

**Published:** 2019-06-27

**Authors:** Esmee Vringer, Stephen W. G. Tait

**Affiliations:** ^1^Cancer Research UK, Beatson Institute, Glasgow, United Kingdom; ^2^Institute of Cancer Sciences, University of Glasgow, Glasgow, United Kingdom

**Keywords:** mitochondria, cell death, inflammation, interferon, NF-κB, apoptosis, caspases, mtDNA

## Abstract

Mitochondrial outer membrane permeabilization (MOMP) is essential to initiate mitochondrial apoptosis. Due to the disruption of mitochondrial outer membrane integrity, intermembrane space proteins, notably cytochrome *c*, are released into the cytosol whereupon they activate caspase proteases and apoptosis. Beyond its well-established apoptotic role, MOMP has recently been shown to display potent pro-inflammatory effects. These include mitochondrial DNA dependent activation of cGAS-STING signaling leading to a type I interferon response. Secondly, via an IAP-regulated mechanism, MOMP can engage pro-inflammatory NF-κB signaling. During cell death, apoptotic caspase activity inhibits mitochondrial dependent inflammation. Importantly, by engaging an immunogenic form of cell death, inhibiting caspase function can effectively inhibit tumorigenesis. Unexpectedly, these studies reveal mitochondria as inflammatory signaling hubs during cell death and demonstrate its potential for therapeutic exploitation.

## Introduction

Mitochondrial outer membrane permeabilization (MOMP), induced by the pro-apoptotic Bcl-2 proteins BAX and BAK, is the essential step in initiating mitochondrial apoptosis. Following MOMP, soluble mitochondrial intermembrane space proteins including cytochrome *c*, SMAC (also called DIABLO) and Omi (also called HtrA2), are released into the cytoplasm. In the cytoplasm, cytochrome *c* binds to APAF-1; this leads to APAF-1 conformational changes and oligomerization into a heptameric wheel-like structure called the apoptosome that recruits and activates the initiator caspase-9 (Bratton and Salvesen, 2010). Active caspase-9 cleaves and activates the executioner caspases-3 and -7, leading to widespread substrate cleavage. Caspase activity is essential for the biochemical and morphological hallmarks of apoptosis, leading to rapid cell death that is considered immunosilent (Arandjelovic and Ravichandran, 2015). Nevertheless, cells usually die irrespective of caspase activation upon MOMP, demarcating it as a point of no return (Tait et al., 2014).

While apoptosis is considered a silent form of cell death, mitochondrial dysfunction (that occurs upon MOMP) is associated with inflammatory effects. For instance, mitochondrial dysfunction can lead to cytosolic exposure of several danger-associated molecular patterns (DAMPs), such as mitochondrial DNA (mtDNA) (Shimada et al., 2012; West et al., 2015) and cardiolipin (Tuominen et al., 2006). Moreover, mitochondrial ROS – increased upon disruption of mitochondrial respiratory chain function – can also promote inflammation (Nakahira et al., 2011; Zhou et al., 2011; Zorov et al., 2014). Once exposed to the cytosol, mitochondrial DAMPs are recognized by various adaptor molecules or receptors leading to an inflammatory response (Grazoli and Pugin, 2018). When mtDNA is in the cytosol it can be recognized by cyclic GMP-AMP (cGAMP) synthetase (cGAS), toll-like receptor 9 (TLR9), and the NLRP3 inflammasome (West and Shadel, 2017), of which the latter can also be activated by mtROS (Shimada et al., 2012). Upon MOMP, release of intermembrane space proteins (Liu et al., 1998; Adrain et al., 2001; van Loo et al., 2002) and cytosolic exposure of the inner mitochondrial membrane occurs (McArthur et al., 2018; Riley et al., 2018), enabling mtDAMP exposure during apoptosis. Various studies have shown that activation of apoptotic caspases has an immunosilencing effect during cell death. The anti-inflammatory effects of apoptotic caspases are likely to be pleiotropic; for instance, caspases have been shown to directly cleave and inactivate inflammatory pathway components as well as strongly suppress protein translation (Clemens et al., 2000; Ning et al., 2019). At least two parallel inflammatory pathways are activated during caspase-independent cell death (CICD) (Rongvaux et al., 2014; White et al., 2014; Giampazolias et al., 2017; McArthur et al., 2018; Riley et al., 2018). In this minireview, we will discuss how MOMP induces inflammation, focusing primarily on two recently described mechanisms: MOMP-induced cGAS-STING signaling (Rongvaux et al., 2014; White et al., 2014; Giampazolias et al., 2017; McArthur et al., 2018; Riley et al., 2018) and activation of pro-inflammatory NF-κB signaling (Giampazolias et al., 2017).

## Mitochondrial Release of mtDNA Causes A Type I Interferon Response

When pathogen-derived, cellular or mitochondrial DNA is present in the cytosol various immunogenic pathways are activated. One of these cytosolic DNA sensors is cGAS, which produces cGAMP, from ATP and GTP, upon DNA binding. cGAMP functions as a secondary messenger and binds to the endoplasmic reticulum (ER) membrane adaptor STING (Cai et al., 2014). Upon binding, STING changes its conformation and becomes activated. Active STING translocates from the ER to an ER-Golgi intermediate apparatus and the Golgi compartment. During this process, the carboxyl terminus of STING recruits and activates TBK1, which in turn phosphorylates the transcription factor IRF3. Phosphorylated IRF3 dimerises and translocates to the nucleus where it initiates a type I interferon response (Chen et al., 2016). The type I interferon response acts in a pleiotropic manner to activate both innate and adaptive immunity (Trinchieri, 2010).

**FIGURE 1:**
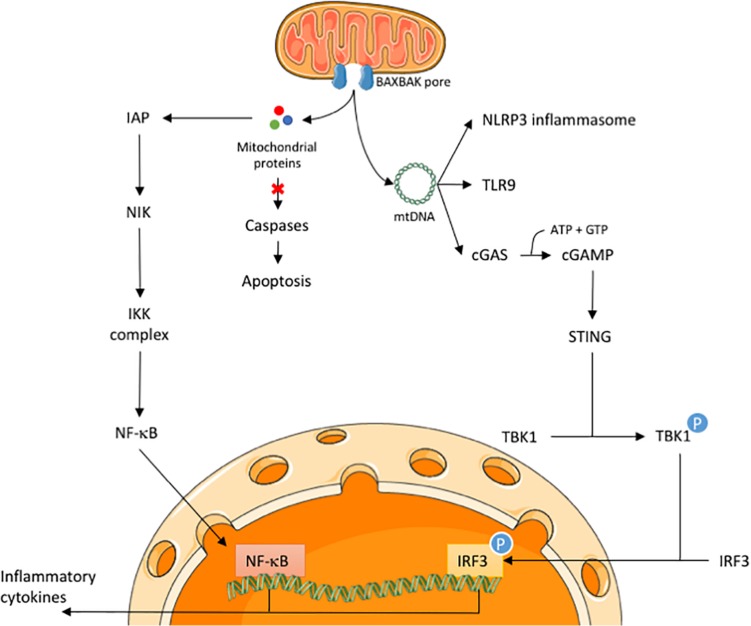
Release of mitochondrial proteins and mtDNA initiates an inflammatory response in CICD. Upon an apoptotic trigger, BAX and BAK form pores in the mitochondrial membrane to allow the release of mitochondrial proteins, such as cytochrome *c* and SMAC/DIABLO, and mtDNA. During CICD, the release of mitochondrial proteins activates the NF-κB pathway via a SMAC-like mechanism. In parallel, release of mtDNA may activate the NLRP3 inflammasome, TLR9 receptor and the cGAS-STING pathway. Activation of the latter will lead to nuclear translocation of IRF3 during CICD. Both NF-κB and IRF3 engage the transcription of various inflammatory cytokines, leading to immune cell activation.

Several years ago, it was found that during mitochondrial apoptosis under caspase-inhibited conditions a type I interferon response is activated (Figure 1; Rongvaux et al., 2014; White et al., 2014). Genetically engineered mouse models and corresponding mouse embryonic fibroblasts with deleted caspases-3 and -7, or -9 showed significantly upregulated type I interferon expression and interferon-stimulated gene response following MOMP. Consistent with this, cells were highly resistant to infection by RNA and DNA viruses (Rongvaux et al., 2014). Similar results were obtained in hematopoietic stem cells, as deletion of caspase 9 increased basal levels of type I interferons and cell death in the presence of caspase inhibition stimulated expression of type I interferons (White et al., 2014). Both groups established that this increase in type I interferons during cell death was due to recognition of mtDNA by cGAS and subsequently STING activation (Rongvaux et al., 2014; White et al., 2014).

During mitochondrial apoptosis, only the outer mitochondrial membrane was thought to permeabilise following BAX and BAK activation. This made it challenging to reconcile how matrix localized mtDNA could activate cytosolic cGAS-STING signaling. Toward answering this conundrum, recent studies have shown that subsequent to MOMP, the inner mitochondrial membrane is extruded through expanding, BAX/BAK-dependent, outer membrane pores (McArthur et al., 2018; Riley et al., 2018; Ader et al., 2019). In the cytosol, these mitochondrial herniations can rupture, enabling mtDNA release (McArthur et al., 2018; Riley et al., 2018). Beyond providing a mechanism for mtDNA dependent activation of cGAS-STING signaling these studies demonstrate that BAX and BAK can form huge, expanding pores, termed macropores (McArthur et al., 2018), on the mitochondrial outer membrane. Previous studies have shown that BAX and BAK pores are highly flexible and dynamic in their pore size and shape in order to release proteins into the cytosol (Bleicken et al., 2013; Große et al., 2016; Salvador-Gallego et al., 2016), however, no study had shown before that these pores could be big enough to release mtDNA from the mitochondria. It is unclear beyond the requirement for BAX/BAK activation whether the release of mtDNA is regulated, but it is independent of both mitochondrial dynamics and mitochondrial permeability transition (Riley et al., 2018).

Besides STING activation, other immune sensing pathways can also be activated by mtDNA. Many antigen presenting cells possess TLR9, which is able to recognize mtDNA by virtue of its high content of CpG rich domains. It has been observed that recognition of mtDNA by TLR9 can both lead to NF-κB translocation and a type I interferon response (Oka et al., 2012; Zhang et al., 2014; Saito et al., 2018). An immune response can also be provoked by activation of the NLRP3 inflammasome via mtDNA during cell death (Nakahira et al., 2011; Shimada et al., 2012; Vince et al., 2018). Activation of the NLRP3 inflammasome leads to processing of interleukin (IL) 1β and IL-18, thereby activating monocytes, macrophages, neutrophils, and T-cells (Netea et al., 2010). Nevertheless, the contribution of these two pathways in the immune response following CICD awaits further investigation.

## Activation of Pro-Inflammatory NF-κB Signaling Following MOMP

In addition to increased expression of type I interferon genes during CICD, we also observed nuclear translocation of NF-κB, thereby activating transcription of pro-inflammatory genes (Giampazolias et al., 2017). NF-κB has been described as having a key role in inflammation, and can be activated in a canonical and non-canonical manner (Lawrence, 2009). Besides inducing the transcription of various inflammatory genes, NF-κB also regulates the activation, differentiation, and effector function of inflammatory T-cells (Lawrence, 2009; Liu et al., 2017).

One of the genes that is transcribed by NF-κB activation is tumor necrosis factor (TNF). TNF is a pro-inflammatory cytokine that can trigger necroptosis, which is a regulated form of cell death that shares morphological similarities with necrosis. It has been observed that CICD has necroptotic features, as the kinetics of cell death were slowed by genetic alteration or pharmacological inhibition of the necroptotic pathway. Inhibiting TNF signaling with Enbrel showed a decrease in cell death, indicating that TNF is needed to engage necroptosis in CICD (Giampazolias et al., 2017). TNF is a well-known activator of NF-κB (Schütze et al., 1992), however, neither TNF nor the necroptotic pathway is responsible for the activation of NF-κB during CICD. Rather, we found that the increase in TNF expression and NF-κB activation during CICD is wholly dependent on BAX and BAK, indicating that MOMP is essential for initiating the inflammatory response. Because of the observation that MOMP is needed to initiate nuclear translocation of NF-κB in caspase inhibited conditions, we can conclude that necroptosis is not essential for the activation of this pathway but accelerates cell death following TNF expression (Giampazolias et al., 2017).

While NF-κB is robustly activated following MOMP, how this is initiated is unclear. A logical explanation may relate to the release of intermembrane space protein SMAC. When SMAC is present in the cytosol it binds to inhibitor of apoptosis proteins (IAPs) to block their function (Du et al., 2000; Verhagen et al., 2000). Besides the role of IAPs to regulate caspase activity and apoptosis, it is also known that IAPs are able to modulate inflammatory signaling by engaging pro-survival NF-κB activation (Gyrd-Hansen and Meier, 2010). Our lab has shown that upon MOMP, NF-κB becomes activated through a SMAC-like mechanism, whereby IAPs are degraded, leading to the activation of NF-κB-inducing kinase (NIK) (Figure 1). Degradation of IAPs occurs in a SMAC-like manner since a non-SMAC binding XIAP mutant is stabilized (Giampazolias et al., 2017). Nevertheless, even in the absence of known intermembrane space IAP-binding proteins, SMAC and Omi, IAPs are degraded following MOMP (Giampazolias et al., 2017). These results demonstrate that neither SMAC or Omi are required for IAP degradation upon MOMP and that some other factor(s) downregulate IAPs in their absence. Interestingly, recent studies have shown that IAP-depletion following MOMP also contributes to caspase-8 dependent inflammasome activation in macrophages (Chauhan et al., 2018; Vince et al., 2018). MOMP-dependent IAP depletion can therefore promote inflammation by at least two distinct pathways.

The question remains as to how IAP degradation is engaged following MOMP. Are proteins in the mitochondrial intermembrane space responsible for this? Or does it relate to the permeabilization of the mitochondrial inner membrane, leading to the release of proteins from the matrix and the inner mitochondrial membrane into the cytosol? In order to fight microbial pathogens, the NF-κB pathway is commonly used and pathogens often interfere with this pathway to escape the immune response (Rahman and McFadden, 2011). Possibly stemming from their bacterial ancestry, could it be that mitochondria have similar pathogen associated molecular patterns (PAMPs) as invading bacteria, and caspases are capable of silencing the mitochondrial PAMPs that activate the NF-κB pathway?

## Cell Death Associated Inflammation: Disease Relevance and Open Questions

In this mini-review we have described how MOMP can lead to an inflammatory response in two parallel pathways. MOMP allows mtDNA to be released into the cytosol, which will be recognized by cGAS leading to activation of STING. This allows phosphorylation of IRF3 to occur, inducing a type I interferon response. In parallel, MOMP leads to the exposure of factors into the cytosol causing degradation of IAPs in a SMAC-like manner. IAP downregulation activates NIK and subsequently NF-κB activation, leading to the transcription of various cytokines. Several questions remain outstanding: is mtDNA release regulated? What triggers NF-kB activation during CICD? How does caspase activity silence inflammation? Does the immune response only occur in caspase-inhibited conditions during cell death? Most importantly, what are the biological roles of these inflammatory effects during apoptotic cell death?

Although apoptosis is a common cell death mechanism during embryonic development, the expression of many pro-apoptotic proteins is reduced in various adult tissues (Sarosiek et al., 2017). Cardiomyocytes for example do not express APAF-1 (Sanchis et al., 2003), thereby being unable to form the apoptosome following MOMP. As a consequence, caspase activation by cytochrome *c* is strictly regulated by endogenous XIAP (Potts et al., 2005). Although cell death is strongly regulated in cardiomyocytes, apoptosis and necrosis does occur in hearts when reperfused after myocardial infarction (MI) (Zhao et al., 2000). Inhibition of caspases in myocardial ischemia/reperfusion-induced models has shown to limit infarct size and to improve recovery in rabbit and rat hearts (Holly et al., 1999; Mocanu et al., 2000; Kovacs et al., 2001). Even when inhibition of caspases does not fully prevent cardiomyocytes from dying, the observed clinical improvement when reperfusion is performed in combination with caspase inhibition suggests that caspase activity negatively impacts recovery via an unknown mechanism. It is well established that during ischemia and reperfusion injury an inflammatory response is provoked by DAMPs derived from necrotic cells in the core of the infarct site. These DAMPs help to recruit leukocytes to the injured areas for cell debris clearance before heart remodeling can take place (Ong et al., 2018). In contrast to the necrotic core of the infarct, apoptosis is primarily observed in the border zone of the infarct (Cheng et al., 1996; Sarate et al., 1997; Toyoda et al., 1997). It has been speculated that loss of cells in the border zone has negative impact on ventricular remodeling (Balsam et al., 2005). Inhibition of caspases might enhance survival of cardiomyocytes in the border zone when the extrinsic apoptotic pathway is activated, however, when MOMP occurs in these cells the intrinsic apoptotic pathway will still lead to cell death in the presence of caspase inhibitors. Nevertheless, engaging a pro-inflammatory response in the border zone through the release of DAMPs in CICD might have beneficial effects. Especially DAMP-associated TNF upregulation during CICD might play a big role in preventing cell loss in the border zone, as a 50% increase in apoptotic cell death was observed when a TNF inhibitor was administered in a mice suffering from MI (Wang et al., 2018). On the other hand, there is evidence that a type I interferon response, which can potentially be induced by the release of mtDNA during MI (Wang et al., 2015), might be harmful as inhibiting this pathway improves cardiac function and ventricular dysfunction after MI (King et al., 2017). This indicates that a fine balance in pro-inflammatory pathways is needed to have a beneficial effect in MI therapy.

In the context of cancer therapy, it has been described in multiple studies that apoptosis can have a pro-tumorigenic effect instead of being anti-tumorigenic (Ichim and Tait, 2016; Cao and Tait, 2018). One of the implications is that if apoptosis is not executed properly, it can cause DNA damage and genomic instability thereby promoting tumor growth (Lovric and Hawkins, 2010; Ichim et al., 2015; Liu et al., 2015). In contrast, engaging CICD in subcutaneously injected tumors in mice causes 50% of the tumors to completely regress. These tumors showed increased expression of cytokines and T-cell infiltration, which was completely reversed when T-cells were depleted in mice, suggesting that the immune system is of great importance in tumor clearance (Giampazolias et al., 2017). Specific inhibition of pro-apoptotic caspases improves cancer cell death by recruiting immune cells to the tumor site. This shows that there is a potential to significantly improve cancer therapy by changing the way cells die from an immunosilent to immunogenic phenotype.

## Author Contributions

Both authors contributed to the research, writing, and editing of the manuscript.

## Conflict of Interest Statement

The authors declare that the research was conducted in the absence of any commercial or financial relationships that could be construed as a potential conflict of interest.
